# Analysis of Simulated Fluorescence Intensities Decays by a New Maximum Entropy Method Algorithm

**DOI:** 10.1007/s10895-012-1135-0

**Published:** 2012-10-19

**Authors:** Rosario Esposito, Carlo Altucci, Raffaele Velotta

**Affiliations:** Dipartimento Scienze Fisiche, Complesso Universitario MonteSantangelo, Universitá di Napoli ‘Federico II’, Naples, Italy

**Keywords:** Maximum Entropy Method, Fluorescence lifetime distributions, Synthetic data

## Abstract

A new algorithm for the Maximum Entropy Method (MEM) is proposed for recovering the lifetime distribution in time-resolved fluorescence decays. The procedure is based on seeking the distribution that maximizes the Skilling entropy function subjected to the chi-squared constraint *χ* ^2^ ~ 1 through iterative linear approximations, LU decomposition of the Hessian matrix of the lagrangian problem and the Golden Section Search for backtracking. The accuracy of this algorithm has been investigated through comparisons with simulated fluorescence decays both of narrow and broad lifetime distributions. The proposed approach is capable to analyse datasets of up to 4,096 points with a discretization ranging from 100 to 1,000 lifetimes. A good agreement with non linear fitting estimates has been observed when the method has been applied to multi-exponential decays. Remarkable results have been also obtained for the broad lifetime distributions where the position is recovered with high accuracy and the distribution width is estimated within 3 %. These results indicate that the procedure proposed generates MEM lifetime distributions that can be used to quantify the real heterogeneity of lifetimes in a sample.

## Introduction

Fluorescence spectroscopy is a classical method for studying the structural and dynamical aspects of biological systems such as proteins, membranes and living cells [1–3]. The excited states of the fluorophores have indeed lifetimes in the range of a few picoseconds to some tens of nanoseconds. Since this corresponds to the time scale of many important biological processes, such as the protein folding dynamics, the protein-DNA interactions, the diffusion of small molecules, the rotational and internal motions, the proton transfer and the energy transfer mechanism [4–6], the time resolved fluorescence has became an important investigation tool.

Despite the great advances in measurement techniques over the recent years, the analysis of fluorescence decays belongs to the difficult tasks in data analysis. Indeed, a parametric multi-exponential model function is usually fitted with the experimental data by non linear least squares methods [7]. Besides a difficulty to find the global overall minimum of the chi-squared function *χ* ^2^ by the parametric minimization procedure, this statistical criteria very rarely allowed to fit the analysed decay curve by more than three exponential functions [4]. Moreover, there are many situations where the time resolved fluorescence profile is described by a continuous lifetime distribution rather than a discrete number of exponential decays. For example, the tryptophan residues in the exited state are usually involved in a energy transfer process to intra-molecular acceptors that depends on the relative distance and affects the fluorescence lifetime [4, 8]. This behaviour of the tryptophan is used to get structural informations on the intermediate forms of proteins that characterise their folding process from an extended random polypeptide chain. In complex situations, such as those encountered even in small proteins, the rate of energy transfer and thus the intra-molecular distances can be described by a continuum distribution due to heterogeneity of the structure that turns out to a continuum lifetime distribution for the fluorescence decay [9–12]. In these cases, the analysis for recovering the distribution takes advantages of a “regularizing function” in addition to the chi-squared statistic for forcing the data to choose one member out of the set of the feasible distributions [13]. The Skilling entropy *S* is one of these regularizing functions and it is maximized subjected to the constraint *χ* ^2^ ~1 by the maximum entropy method (MEM) thus determining the desired distribution with no assumptions about its functional form [14–21].

The MEM is a mathematically complex algorithm that seeks the stationary point of the Lagrange function Λ = *S* + *λ*(*χ* ^2^ − 1), *λ* being the so-called Lagrange multiplier, that turns out to be the solution of the set of non linear equations resulting from $\nabla \Lambda=0$. Due to the once limited computer memory and low performing computational time, the conjugate gradient method (CG) [22] has been the iterative method widely spread for the solution of equation systems [23]. The necessary compromise between the accuracy of the solution and the convergence speed of the algorithm [24] limited both the number of data points and the number of lifetimes used for reconstructing the desired distribution to a maximum values of about 1,000 and 200 respectively, with the obvious limitation of the information content in the MEM distribution [25, 26].

Nowadays, the extensive computing power of a standard home computer makes possible the adoption of new iterative approaches to the MEM based on direct methods. In this paper we present a new procedure based on the Newton-Raphson method for solving the Lagrange multiplier problem associated to the MEM constrained problem. A method for the solution of the equation system $\nabla \Lambda=0$ through iterative linear approximations and LU decomposition [27] is extensively discussed. The implementation of the MEM is carried out using a homebuilt routine package written in MATLAB (version 7.14, The MathWorks Inc., Natick, MA, 2012). The accuracy of the algorithm is investigated through comparisons with numerical simulations of fluorescence decay data. It results that the MEM algorithm proposed can analyse datasets with up to 4,096 data points, that is a typical value of an experimental set up based on a time-correlated single photon counting technique, by considering distributions with a number of lifetimes that ranges from 100 to 1,000.

## Theory of the Method

The time-resolved fluorescence decay data $\lbrace E_m \rbrace$ can be described by a function *T*(*t*) defined as a discrete convolution product of the intensity decay function *I*(*t*) by the impulse response function *R*(*t*):

1 $$ T(t) = \sum\limits_{m=1}^{M} {R(t_m) \cdot I(t-t_m)} \label{T} $$

with *t*_*m*_ the measurement time of the m-th data point *E*_*m*_ and *M* the number of data points. The quantity *R*(*t*_*m*_) is experimentally measured at each time *t*_*m*_ whereas *I*(*t*) is usually modelled as a discrete sum of exponential decays [28]:

2 $$ I(t) = \sum\limits_{k=1}^{N} \alpha_k \rm{e}^{-\frac{t}{\tau_k}}, \label{i_2} $$

For the purpose of numerical calculations the sum in Eq. 2 is extended from the resolution limit *τ*_1_ imposed by the experimental set-up to the maximum lifetime *τ*_*N*_ of the chromophore observed. The quantity *N* is the number of partitions of the interval [*τ*_1_, *τ*_*N*_], *α*_*k*_ and *τ*_*k*_ the amplitude factors and the lifetime of the k-th partition, respectively. The expression 2 allows to write the convolution sum expressed by the Eq. 1 as follows:

3 $$ \begin{array}{rll} T(t_m) &=& \sum\limits_{k=1}^N {C_{m,k} \cdot \alpha _k} \\ C_{m,k} &=& \sum\limits_{i=1}^M {R(t_i}){\rm{e}^{ - \frac{{t_m - t_i}}{\tau_k}}} \label{T_2} \end{array} $$

There are many sets $\lbrace\alpha_k\rbrace$ physically allowable that agree with the data within the experimental precision $\lbrace \sigma_k \rbrace$ [15] and the Maximum Entropy Method selects the distribution $\lbrace\alpha_k\rbrace$ that maximizes the Skilling entropy function *S* [14]

4 $$ S(\alpha_{1},...,\alpha_N) = \sum\limits_{k=1}^{N} {\alpha_k - \alpha_k \log(\alpha_k) } \label{entropy} $$

subjected to the following chi-squared condition:

5 $$ \chi^{2} = \frac{1}{M} \sum\limits_{m=1}^{M} \frac{\left(E_{m}-T(t_m)\right)^2}{\sigma_m^2 } \sim 1 \label{chi} $$

with the lifetime components equally spaced in a logarithmic scale. Note that in Eq. 5, the number *M* of observations is not reduced by the number *N* of parameters, because this condition is not used to find the optimal set of parameters in *α*-space but it is used to test whether or not computed data is in satisfactory agreement with measured data [15].

In the following a new algorithm for maximizing the entropy *S* according to the MEM requirements will be described. The procedure refers to the negative function − *S* which is minimized by the same distribution that maximizes *S* obviously.

The method of Lagrange multiplier provides a strategy for finding the minimum of -S with the constraint 5 by considering the following Lagrange function

6 $$ \Lambda\left(\alpha_1,...,\alpha_N, \lambda\right) = -S\left(\alpha_1,...\alpha_N\right) + \lambda \cdot \left(\chi^2 -1\right), \label{funcional} $$

where *λ* is the Lagrange multiplier.

The MEM solution is an extremal of Λ for some value of the Lagrange multiplier *λ*, thus it is obtained by solving the set of non linear equations $\nabla \Lambda = 0$:

7 $$ \begin{array}{rll} && (\nabla \Lambda)_{i} = [\nabla (-S)]_{i} + \lambda \left(\nabla \chi^2\right)_{i} = 0 \qquad \;\; i = 1..N \\ && (\nabla \Lambda)_{N+1} = \chi^2 -1 = 0 \label{set} \end{array} $$

with

8 $$ \begin{array}{rll} [\nabla (-S)]_{i} &=& \log(\alpha_i); \\ (\nabla \chi^2)_{i} &=& - \frac{2}{M} \sum\limits_{m=1}^{M} \frac{\left(E_m-\sum_{k=1}^N C_{m,k} \alpha_k\right)}{\sigma_m^2 }C_{m,i} \label{log} \end{array} $$

The subscript *i* is used to indicate the first N components of gradients $\nabla \Lambda$, $\nabla (-S)$ and $\nabla \chi^2$, whereas the last component is given by the subscript *N* + 1.

Now let **x** denote the vector of components (*α*_1_,...,*α*_*N*_, *λ*) and $\textbf{F} = \nabla \Lambda$. In order to solve the set 7, each of the equations *F*_*i*_ = 0 is expanded in Taylor series:

9 $$ F_{i}(\mathbf{x} + \delta \mathbf{x}) = F_{i}(\mathbf{x}) + \sum\limits_{j=1}^{N+1} {\frac{\partial F_i} {\partial x_j} \delta x_{j} + O\left(\delta \mathbf{x^2}\right)} \label{eqscal} $$

The matrix of partial derivatives appearing in Eq. 9 is the Hessian matrix *H* of the function Λ:

10 $$ H_{i,j} = \frac{\partial F_i}{\partial x_j} = \frac{\partial^{2} \Lambda}{\partial x_j \partial x_i} $$

and its elements are given by:

$$ \begin{array}{llr} \frac{\partial^2 \Lambda}{\partial x_j \partial x_i} = \frac{\partial^2 \Lambda}{\partial x_{i} \partial x_{j}} = \frac{\delta_{i,j}}{x_j} + \frac{2 \lambda}{M} \sum\limits_{m=1}^{M} \frac{C_{m,i} C_{m,j}}{\sigma^2_m},&& \\&& i,j \leq N \end{array} $$

11 $$ \begin{array}{rll} \frac{\partial^2 \Lambda}{\partial x_{N+1} \partial x_i} &=& \frac{\partial^2 \Lambda}{\partial x_i \partial x_{N+1}} = -\frac{2}{M} \\ &&\times \sum_{m=1}^{M} \frac{\left(E_m-\sum_{k=1}^N C_{m,k} x_k\right)}{\sigma_m^2 } C_{m,i}, \\ &&\quad\qquad\qquad\qquad\qquad i \leq N, j = N+1 \\ \frac{\partial^2 \Lambda}{\partial x_{N+1} \partial x_{N+1}} &=& 0 \qquad\qquad\qquad i,j=N+1 \end{array} $$

In matrix notation the Eq. 9 is

12 $$ \mathbf{F}(\mathbf{x} + \delta \mathbf{x}) = \mathbf{F}(\mathbf{x}) + H \cdot \delta \mathbf{x} + O\left(\delta \mathbf{x^2}\right) \label{eqvect} $$

By neglecting terms of order *δ* **x** ^**2**^ and by setting **F**(**x** + *δ* **x**) = 0, we obtain a set of linear equations for the corrections *δ* **x** (the Newton step), namely

13 $$ H \cdot \delta \mathbf{x} = - \mathbf{F} \label{apprlin} $$

The solution to the set of equations 7 is obtained by solving iteratively the linear set 14 that minimizes the norm *f* = 1/2 **F** ·**F** because the correction *δ* **x** move each function *F*_*i*_ closer to zero simultaneously at each iteration. The first iteration is performed by considering the distribution $\lbrace\alpha_k\rbrace$ that equals the calculated total intensity to the experimental one, namely a flat distribution with a constant value of ∑ _*m*_ *E*_*m*_/ ∑ _*m*,*k*_ *C*_*m*,*k*_ for all lifetimes.

The matrix equation 13 is solved by the package *linsolve* of Matlab that implements a LU decomposition algorithm that writes H as a product of two matrices [27]:

14 $$ H = L \cdot U $$

where *L* and *U* are respectively a lower and upper triangular matrix. Their elements are obtained by the Crout’s algorithm whose stability is increased by a partial pivoting technique [27]. The decomposition is used first for solving for the vector **y** such that

15 $$ L \cdot \mathbf{y} = - \mathbf{F} $$

and then for solving

16 $$ U \cdot \delta \mathbf{x} = \mathbf{y} $$

The advantage of breaking up the linear set 14 into two successive ones is that the solution of a triangular set of equations is quite trivial and is given by back-substitution [29]. Once the new approximated solution **x** + *δ* **x** is obtained, one seeks negative values among its first N components because the natural logarithm in Eq. 8 requires only positive values. If this is the case, the positiveness is enforced by using only a fraction of the corrections *δ* **x**.

Generally, the Newton step *δ* **x** is a descent direction for the norm *f*:

17 $$ \nabla f \cdot \delta \mathbf{x} = (\mathbf{F} \cdot H) \cdot (-H^{-1} \cdot \mathbf{F}) = - \mathbf{F} \cdot \mathbf{F} < 0 $$

Therefore, we always first try the full correction *δ* **x** because once we are close enough to the solution a quadratic convergence is given and the proposed step reduces *f*. Conversely, if the linear approximation 12 is not satisfied, *δ* **x** need not decreases the norm *f* and a backtrack along the Newton direction is adopted until an acceptable step is obtained. Particularly, one moves to a new point

18 $$ \mathbf{x_{new}} = \mathbf{x} + \epsilon \delta \mathbf{x},~~~0 < \epsilon \leq 1 $$

and the parameter *ε* is chosen to minimize f in the direction *δ* **x** . This goal is achieved by using the routine *fminbnd* of Matlab that implements the Golden Section Search algorithm [30]. The improved approximation **x**_**new**_ is used as a new starting point for the set of equations 14 and the procedure is iterated until the minimum of the functional Λ is achieved. The set of values $\lbrace\alpha_k\rbrace$ that minimizes Λ with *χ* ^2^ ~1 is the lifetime distribution that accounts for the experimental data.

## Analysis of the Accuracy of the MEM Algorithm

In the following the accuracy of the MEM algorithm proposed in the previous section is investigated through comparisons with numerical simulations of fluorescence decay data. Synthetic data $\lbrace E_m \rbrace$ are generated with time scale and time resolution that are typical in experimental set-up for time-correlated single photon counting technique (TCSPC) [31]. The simulated curves consist of 4,096 data points over a time scale of 25 ns and are generated by the convolution product of a Gaussian profile with full-width at half maximum (FWHM) of 120 ps, the impulse response function *R*(*t*), and a decay model function according to the Eq. 3. The Poisson noise statistics that affects the typical TCSPC measurements [32] is simulated by taking as a value for the intensity at each time a Poisson-distributed random number with a mean equal to the calculated model value *E*_*m*_. The routine *poissrnd* of Matalb is used to this purpose and its algorithm is extensively described in [33]. According to this procedure for generating simulated data, the number of counts in each point is its variance *σ* ^2^ and the peak counts can be considered as a measure of the noise content of the decay curve.

### Multi-Exponential Fluorescence Decays

**Fig. 1:**
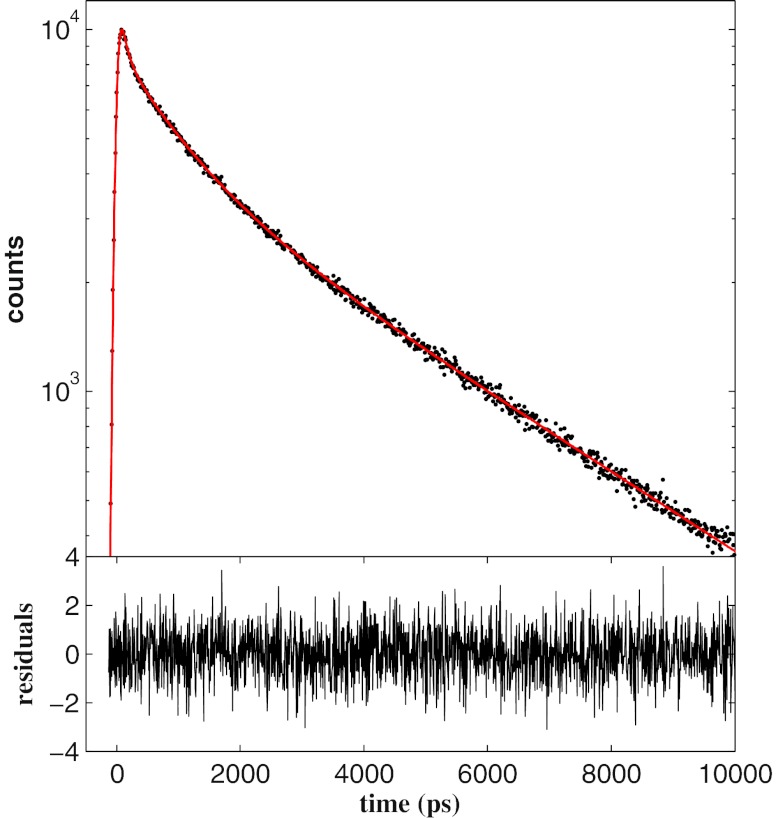
The fluorescence decay intensity (*black points*) simulated by three exponential functions with lifetimes *τ*_1_ = 100 ps, *τ*_2_ = 1,000 ps and *τ*_3_ = 4,000 ps and the same value for the relative amplitude *α*_*j*_ = 0.33. The *red solid line* is the curve fitted with the MEM for *N* = 400 exponential decays. The residuals are reported in the *lower panel*

**Fig. 2:**
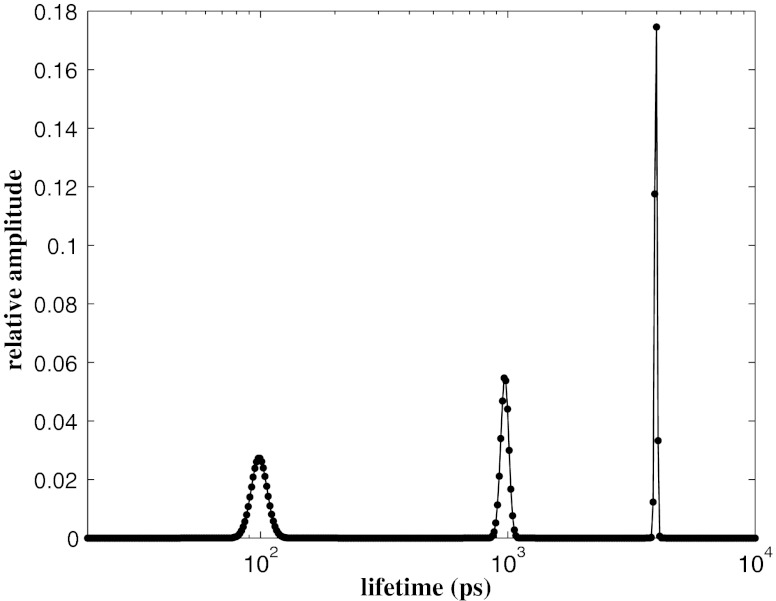
The normalised lifetime distribution *α*(*t*) / ∑ *α*(*t*)Δ obtained by the MEM analysis of a simulated noisy multi-exponential decay with 10^4^ counts in the peak channel. The simulated data consists of 4,096 data points on a time scale of 25 ns and are characterised by 3 lifetimes: *τ*_1_ = 100 ps, *τ*_2_ = 1,000 ps and *τ*_3_ = 4,000 ps. The MEM results are obtained for *N* = 400 points equally spaced in log*τ* between 20 ps and 10^4^ ps and are reported in Table 1

Multi exponential intensity decays are usually adopted for the analysis of the fluorescence emission of proteins in aqueous solutions and lie on the border of complexity that can be handled by parametric methods. In Fig. 1 we report a fluorescence decay curve (black points) with 10^4^ counts in the maximum generated by three exponential functions with lifetimes *τ*_1_ = 100 ps, *τ*_2_ = 1,000 ps and *τ*_3_ = 4,000 ps and amplitude *α*_*j*_ = 0.33, *j* = 1,...,3. The solid red line is the curve resulting from the fitting with the MEM algorithm by considering *N* = 400 points equally spaced in log*τ* between *τ*_min_ = 20 ps and $\tau_{\rm max}=10^4$ ps. The MEM convergence is achieved for a chi-square value equals to *χ* ^2^ = 1.01 and the accuracy of the algorithm is confirmed by the random distribution of the residuals around zero as it is shown in the lower panel of Fig. 1. The normalised lifetime distribution that accounts for the agreement between the MEM curve and the synthetic decay data is reported in Fig. 2. The distribution consists of multiple peaks whose mean position $\langle \tau_j \rangle$ and mean amplitude $\langle \alpha_j \rangle$ are calculated as follows

19 $$ \begin{array}{rll} \langle \tau_j \rangle &=& \frac{\sum_{k=1}^{N_j} \alpha_k \tau_k \Delta_k}{\sum_{k=1}^{N_j} \alpha_k \Delta_k} \nonumber \\ \langle \alpha_j \rangle &=& \frac{\sum_{k=1}^{N_j} \alpha_k \Delta_k}{\sum_{k=1}^{N} \alpha_k \Delta_k} \label{average} \end{array} $$

where *N*_*j*_ is the number of lifetimes represented in each peak and Δ_*k*_ is the spacing in log*τ*. The calculated mean values $\langle \tau_j\rangle$ and $\langle \alpha_j \rangle$ are reported in Table 1 and are the retrieved lifetimes and the pre-exponential factors of the analysed decay curve. Similarly, the standard deviation Δ*τ* of the data points of the peak region is considered as the uncertainty in the lifetime estimate, whereas the error of the amplitude factor is given by the standard deviation of the values calculated for a set of 20 simulated decay curves. The parameters generated by a non linear least-square regression analysis with a three exponential model function are also shown in the Table 1 for comparison. It results a very good agreement among the estimates of the amplitudes *α*_*j*_ given by both methods. The values retrieved by the non linear fitting are indeed within three standard deviations and have relative errors lower than 3 %, whereas the relative amplitudes calculated from the MEM lifetime distribution agree with the theoretical values within 2 %. As far as it concerns the three lifetimes, the values of *τ*_1_, *τ*_2_ and *τ*_3_ are recovered by the MEM within one standard deviation, whereas the non linear fitting procedure achieves this accuracy only for *τ*_1_, as *τ*_2_ and *τ*_3_ lie within two and four standard deviations, respectively. The long lifetime *τ*_3_ even outside the “three-sigma” interval witnesses the difficulty in fitting decay curves with more than three exponential functions.

**Table 1:** The decay parameters recovered by the MEM analysis of a three exponential decay with 10^4^ counts in the peak channel

Simulated parameters	*α*_1_	*α*_2_	*α*_3_	*τ*_1_ (ps)	*τ*_2_ (ps)	*τ*_3_ (ps)	
	0.33	0.33	0.33	100	1,000	4,000	

MEM results	$\langle \alpha_1 \rangle$	$\langle \alpha_2 \rangle$	$\langle \alpha_3 \rangle$	$\langle \tau_1 \rangle$	$\langle \tau_2 \rangle$	$\langle \tau_3 \rangle$	*χ* ^2^
	0.33 ±0.01	0.337 ±0.005	0.333 ±0.003	99 ±7	970 ±40	3,970 ±40	1.01

Least-square fit	*α*_1_	*α*_2_	*α*_3_	*τ*_1_ (ps)	*τ*_2_ (ps)	*τ*_3_ (ps)	*χ* ^2^
	0.33 ±0.01	0.332 ±0.006	0.340 ±0.004	99 ±5	970 ±15	3,960 ±10	1.01

The uncertainties of the lifetime estimates $\langle \tau_j \rangle$ are given by the standard deviations *Δτ* of the data points of the peak regions, whereas the errors of the amplitude factors $\langle \alpha_j \rangle$ are the standard deviations of the values calculated for a set of 20 synthetic curves. Results of non linear least-square regression analysis are also reported for comparison together with the theoretical values of the simulated curve. In this case the uncertainties of the parameters are given by the fitting procedure

**Fig. 3:**
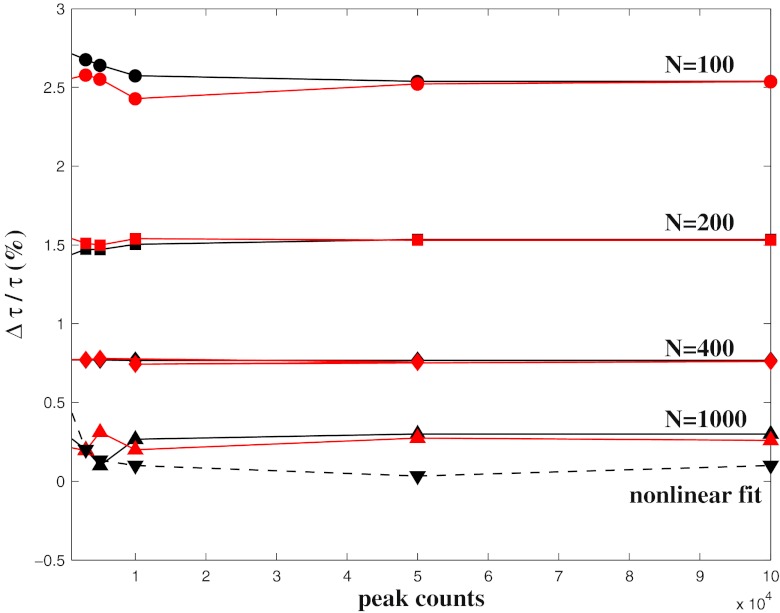
The ratio $r=\Delta \tau / \langle \tau \rangle$ calculated for the MEM lifetime spectrum of a single exponential decay with lifetime *τ* = 3,000 ps as a function of peak counts. The values of *r* have been calculated for different values of the number of lifetimes *N* = 100,200,400,1,000 that ranges from 20 ps to 10^4^ ps. The *black solid curves* are obtained by considering *M* = 4,096 data points in the MEM analysis, whereas the *red solid curves* are given for *M* = 1,024 data points. The *black dotted curve* is the uncertainty of the mean lifetime estimated by the non linear fit

The MEM accuracy in reconstructing a lifetime spectrum *α*(*τ*) can be analysed by considering the ratio $r=\Delta \tau / \langle \tau \rangle$. In Fig. 3 the values of *r* calculated for a single exponential decay with lifetime *τ* = 3,000 ps are reported as a function of peak counts. The results have been obtained for a MEM analysis that has considered both *M* = 4,096 (black solid curves) and *M* = 1,024 (red solid curves) data points. A lifetime spectrum equally spaced in log*τ* between 20 ps and 10^4^ ps has been considered with a discretization that ranges from *N* = 100 to *N* = 1,000 in order to investigate the sensitivity of the method to the number of data points and the lifetimes in log*τ* space, respectively. From inspection of the curves it results the ratio *r* does not depend on the noise level when the number of counts of the peak is larger than 3 − 5×10^4^. The ratio *r* is always smaller than 3 % and decreases as the number N of discretization of the lifetimes space increases. When N is larger than 400 *r* is smaller than 1 % reaching the uncertainty of the non linear fit (black dotted curve) when *N* is larger than 1,000. Furthermore, the results obtained for *M* = 4,096 and *M* = 1,024 data points are comparable showing that the MEM accuracy is not affected by the number of data points for *M* ≥ 1,024.

### Gaussian Lifetime Distributions

**Fig. 4:**
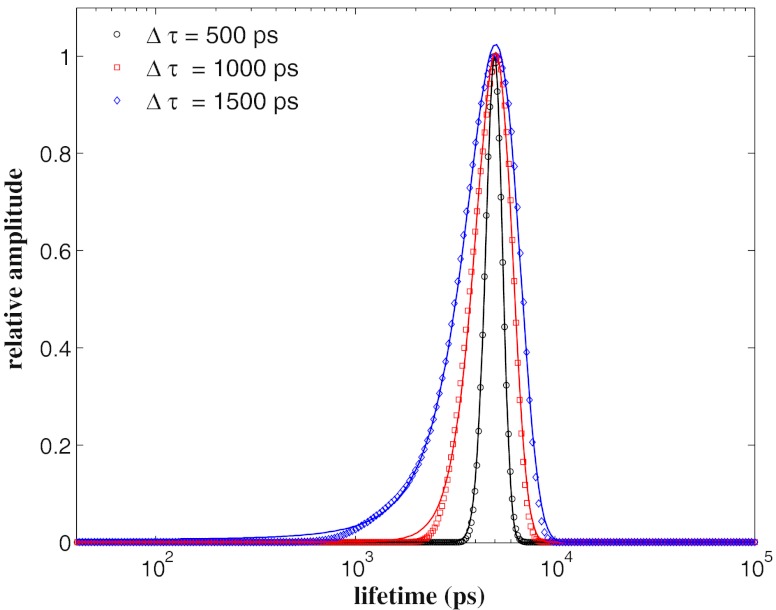
The normalized lifetimes spectra *α*(*τ*) reconstructed by the MEM analysis performed on fluorescence decay intensities with ~5 ×10^4^ counts in the peak channel by using *N* = 400 points equally spaced in log*τ* between 40 ps and 10^5^ ps. The Gaussian distributions are centred at *τ* = 5,000 ps and have different standard deviations, Δ*τ* = 500 ps (*black open circles*), Δ*τ* = 1,000 ps (*red open squares*), Δ*τ* = 1,500 ps (*blue open diamonds*). The *solid lines* are the Gaussian fitted profiles

To test the performances of our algorithm with broad lifetime distributions, we considered Gaussian distributions centred at *τ* = 5,000 ps with different standards deviation Δ*τ* = 500 ps, 1,000 ps, 1,500 ps (i.e. 10, 20 % and the 30 % of *τ*), that are assumed as a measure of their width. A set of 20 fluorescence decays curves with *M* = 4,096 data points and ~5×10^4^ number of counts at the maximum has been generated for each of these distributions. Each curve of every set has been analysed with the MEM by using *N* = 400 points equally spaced in log*τ* between 40 ps and 10^5^ ps and each normalised MEM spectrum *α*(*τ*) has been fitted with a Gaussian profile to estimate the center and the width of the distribution. In Fig. 4 the typical MEM spectra obtained for each set (differently marked points) are shown together with the Gaussian fitted curves (solid lines) whereas the mono-modal section of the Table 2 reports the mean values and the uncertainties of the center and the width Δ*τ* of the Gaussian distributions calculated for each set of curves. The uncertainties are the standard deviations of the estimates of the parameters calculated for each set. Figure 4 shows that the MEM algorithm reproduces the Gaussian profile of the distributions with excellent resolution of the leading and the falling edges. The fitting results are reported in the Table 2, where a good agreement is observed between the values estimated for the parameters of the Gaussian lifetime distributions and the theoretical ones.

**Table 2:** The center and the width Δ*τ* of the Gaussian lifetime distributions recovered by the MEM and estimated through a fitting procedure

	Gaussian 1-modal distribution		Gaussian 2-modal distribution			
	Nominal values	MEM results	Nominal values		MEM results	
Amplitude			1	1	0.92 ±0.02	1
Center (ps)	5,000	4,990 ±15	1,000	5,000	985 ±34	5,016 ±23
Width Δ*τ* (ps)	500	498 ±15	300	1,500	319 ±31	1,515 ±95
Amplitude			0.5	1	0.45 ±0.03	1
Center (ps)	5,000	5,008 ±19	1,000	5,000	989 ±42	5,012 ±24
Width Δ*τ* (ps)	1,000	1,029 ±20	300	1,500	329 ±38	1,528 ±54
Amplitude			0.25	1	0.23 ±0.01	1
Center (ps)	5,000	5,009 ±10	1,000	5,000	990 ±30	5,038 ±24
Width Δ*τ* (ps)	1,500	1,524 ±15	300	1,500	315 ±24	1,546 ±31
Amplitude			0.1	1	0.092 ±0.008	1
Center (ps)			1,000	5,000	1,036 ±87	5,006 ±17
Width Δ*τ* (ps)			300	1,500	336±49	1,509 ±31

A set of 20 fluorescence decay curves with 5 ×10^4^ counts in the peak channel has been generated for each distribution. The reported values are the means and the standard deviations of the estimates of the parameters calculated for each set. The theoretical values are also reported for comparison. The results have been obtained by using *N* = 400 points equally spaced in log*τ* between 40 ps and 10^5^ ps

The resolution limit of the algorithm in resolving the intensities of the lifetimes spectrum has been investigated by adding a Gaussian distribution with different peak intensities on the tail of the distribution peaked at *τ* = 5,000 ps. To this purpose, we have considered the worst case of Fig. 4 observed for a width Δ*τ* = 1,500 ps and added an additional distribution centred at *τ* = 1,000 ps with a width Δ*τ* = 300 ps. Four values for the amplitude of this additional peak have been considered: 100, 50, 25 and the 10 % of the main peak at *τ* = 5,000 ps. As before, a set of 20 fluorescence decay curves has been generated in each case for working out the errors in recovering the characteristic parameters of these bi-modal lifetime distributions through a bi-Gaussian fitting procedure.

**Fig. 5:**
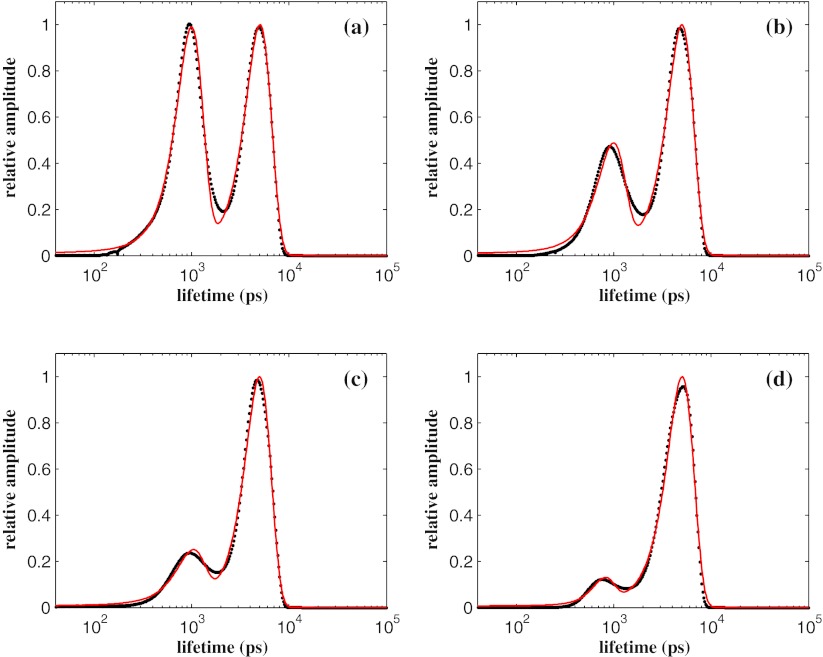
The normalized lifetimes spectra *α*(*τ*) reconstructed by the MEM analysis (*black points*) for a bi-modal Gaussian distribution with the main peak centred at *τ* = 5,000 ps and the secondary to *τ* = 1,000 ps. Four values for the amplitude of the additional peak have been considered and they represent 100 % **a**, 50 % **b**, 25 % **c** and the 10 % **d** of the main peak. Both Gaussians have size equals to the 30 % of their peak location. The results refer to fluorescence decay intensities with ~5 ×10^4^ counts in the peak channel and have been obtained by using *N* = 400 points equally spaced in log*τ* between 40 ps and 10^5^ ps. The solid red line are the two Gaussian fitted profiles used to estimate the characteristic parameters of the bi-modal distribution (see Table 2)

In each panel of the Fig. 5 the typical MEM spectrum (black points) and the best fit (red curve) by two Gaussian curves have been shown. The average for each set of decay curves of the peak position (center), the height (amplitude) and the width (Δ*τ*) are reported in Table 2. As it can be seen, the position and the width of the main peak are estimated with an accuracy that is not affected by the presence of the secondary peak. Similar results are observed for the retrieved parameters of the secondary peak showing that our algorithm resolves two distributions even when their amplitude are in the ratio 1:10.

## Conclusions

We have described a new algorithm for implementing a MEM analysis of the time-resolved fluorescence decays. The proposed procedure is based on seeking the desired lifetime distribution by solving the set of non linear equations $\nabla \Lambda = 0$ through iterative linear approximations, LU decomposition of the Hessian matrix *H* and the Golden Section Search for backtracking. This algorithm has been tested on complex analytically simulated data that arise from multi-exponential decays and from broad lifetime distributions. A typical inversion with *M* = 4,096 data points and *N* = 400 discretization of the lifetime spectrum takes about 60 s in total using an Intel(R) Core(TM)i7-2600 CPU@3.40 GHz processor with 8 GB memory. The computation time is about 150 s when the number of lifetimes considered is *N* = 1,000.

The analysis of the multi-exponential decays with our computational approach has clearly shown that the sensitivity of the MEM increases as the number *N* of discretization in log*τ* space increases. An accuracy in retrieving the simulated parameters comparable to that of non linear regression can be achieved by considering *N* = 1,000 lifetimes.

The characteristic parameters of the broad lifetime distributions have been retrieved with high accuracy. Particularly, our MEM procedure has retrieved the widths Δ*τ* that represent 10 % of the lifetime with a discrepancy lower than 3 % by using a typical value of 5×10^4^ counts in the maximum peak. This result clearly indicates that the procedure proposed generates MEM lifetime distributions that can be used to quantify the real heterogeneity of lifetimes in a sample with no need for a time consuming high statistic. Moreover, the analysis of bi-modal distributions has demonstrated that our algorithm resolves a secondary peak even when its relative amplitude is 10 % of the amplitude of the neighbourhood peak.

It is also important to highlight that the MEM algorithm proposed in this paper extends the analysis to datasets of up to 4,096 data points, thereby increasing the limit currently set at about 1,000. This increases the information that can be achieved from the data thus improving the accuracy in recovering the fast decay times; in fact, short lifetimes affect the leading edge that only contains few points. On the other hand, a larger density (*N* ≥ 1,000) is beneficial also for larger lifetimes since their spectrum can be determined with higher resolution.

### **Open Access**

This article is distributed under the terms of the Creative Commons Attribution License which permits any use, distribution, and reproduction in any medium, provided the original author(s) and the source are credited.
